# Alteration of Brain Structure With Long-Term Abstinence of Methamphetamine by Voxel-Based Morphometry

**DOI:** 10.3389/fpsyt.2018.00722

**Published:** 2018-12-20

**Authors:** Zhixue Zhang, Lei He, Shucai Huang, Lidan Fan, Yining Li, Ping Li, Jun Zhang, Jun Liu, Ru Yang

**Affiliations:** ^1^Department of Radiology, The Second Xiangya Hospital, Central South University, Changsha, China; ^2^Department of Radiology, The First Hospital of Hunan University of Chinese Medicine, Changsha, China; ^3^Department of Psychiatry, The Fourth People's Hospital of Wuhu, Wuhu, China; ^4^Hunan Judicial Police Academy, Changsha, China

**Keywords:** addiction, methamphetamine, long-term abstinence, voxel-based morphometry, gray matter volume

## Abstract

**Background:** A large portion of previous studies that have demonstrated brain gray matter reduction in individuals who use methamphetamine (MA) have focused on short-term abstinence, but few studies have focused on the effects of long-term abstinence of methamphetamine on brain structures.

**Materials and Methods:** Our study includes 40 healthy controls and 44 abstinent methamphetamine-dependent (AMD) subjects who have abstained for at least 14 months. For every AMD subject, the age when they first used MA, the total time of MA use, the frequency of MA use in the last month before abstinence, the duration of abstinence and the craving score were recorded. Here we used magnetic resonance imaging (MRI) to measure the gray matter volume (GMV) of each subject with voxel-based morphometry method. Two-sample *t*-test (AlphaSim corrected) was performed to obtain brain regions with different gray matter volume (GMV) between groups. In addition, partial correlation coefficients adjusted for age, years of education, smoking, and drinking were calculated in the AMD group to assess associations between the mean GMV values in significant clusters and variables of MA use and abstinence.

**Results:** Compared with the healthy control group, AMD group showed increased gray matter volumes in the bilateral cerebellum and decreased volumes in the right calcarine and right cuneus. Moreover, GMV of left cerebellum are positively correlated with the duration of abstinence in the AMD group (*p* = 0.040*, r* = 0.626).

**Conclusions:** The present study showed that the gray matter volume in some brain regions is abnormal in the AMD subjects with long-term abstinence. Changes in gray matter volume of visual and cognitive function regions suggested that these areas play important roles in the progress of MA addiction and abstinence. In addition, positive correlation between GMV of the left cerebellum crus and duration of abstinence suggested that prolonged abstinence is beneficial to cognitive function recovery.

## Introduction

Methamphetamine is a highly addictive psychostimulant drug that principally affects the monoamine neurotransmitter systems of the brain and results in feelings of alertness, increased energy and euphoria (1–3). This drug has become a global public health problem due to its ease of production and has more rapid onset and serious neurotoxic effects compared with other traditional drugs (4, 5). World Drug Report 2018 (6) described that up to 2016, about 31 million drug users have shown to have problematic use of drugs. In China, it is reported that there are 2.5 million people that have problematic use of illicit drugs. Synthetic drugs remain the major source of abuse. Among the various drugs seized, methamphetamine and related products account for 31.6%, which is much higher than 10.6% for heroin and 8.1% for ketamine. As a consequence, methamphetamine abuse and related substances have become a serious health crisis (7). Methamphetamine abuse can cause various physical illnesses and psychotic disorders (8–11). Worse of all, even after undergoing substance abuse treatment, patients often relapse when they encounter stress and other high risk environments that may trigger drug use (12–14).

Researches have shown that methamphetamine abuse causes comprehensive changes to brain structures and functions (15–17). After a period of abstinence, brain structure and metabolism can be restored and improved to a certain extent (18–20). Among various methods to investigate brain changes, voxel-based morphometry (VBM) is an automated and efficient tool for whole-brain analysis to detect structural differences. This method is sensitive to subtle brain alterations in gray matter. VBM was developed to detect group differences in the relative concentration of gray matter tissues across the whole brain in a voxel-wise manner. Comparing with traditional morphometric approaches which rely on measuring brain volumes manually, it provides more rapid results and is used for various brain regions (21). Hence, it has been widely used in studies on psychiatric disorders including chemical substance addiction (22, 23). For example, Hanlon and Canterberry (24) indicated that the duration of abstinence was associated with increased gray matter volume (GMV) in the dorsolateral prefrontal cortex, posterior cingulate cortex, and superior parietal lobe by studying about 40 male cocaine abusers. A study on alcohol abstinent patients has shown that abstinence therapy is beneficial for the recovery of GMV in the frontal lobe (25). However, these studies using the VBM method mainly focused on short-term abstinence and results from various studies are inconsistent (26–28).

To investigate brain structure with long-term abstinence could further illuminate the nature of drug relapse, thus conducive to improve long-term abstinence treatment efficacy and rehabilitation programs. Yang et al. (29) utilized a non-human primate model of addiction and showed that neurochemical changes associated with long-term drug use do not persist after prolonged abstinence, suggesting therapeutic effects of long-term abstinence. Wang et al. (30) found that thalamic metabolism was recovered and was associated with improved performance in motor and verbal memory tasks in five long-term abstinent MA abusers. However, these studies which focused on long-term abstinence are either based on small samples or on non-human primate.

In order to overcome shortcomings of the related studies mentioned above, in this study, relatively larger samples (44 AMD subjects and 40 healthy controls) with a long abstinence duration (14–25 month) were collected to investigate the volume changes on gray matter of AMD patients compared with healthy controls (HC) using the VBM method.

## Materials and Methods

### Subjects

Our study includes 44 AMD subjects and 40 healthy control subjects. All AMD subjects are from Pingtang Mandatory Detoxification in Changsha City, Hunan Province. They all were diagnosed using the Diagnostic and Statistical Manual on Mental Disorders (DSM-V) and after that had received a long-term (14–25 month) compulsory abstinence. During abstinence, the participants were treated with medicine, education, and physical exercise and didn't show any significant abstinence symptoms. Inclusion criteria for all subjects included males, ranging in age from 18 to 45 years old, graduated from at least elementary school, normal visual acuity with or without lens correction, normal hearing, and right handed. Exclusion criteria included diseases that affect cognitive function such as head trauma history, cerebrovascular disease, epilepsy, severe mental illness, severe heart, liver, kidney diseases, drug use in the past 6 months, other substance use or dependence except nicotine in the past 5 years, contraindications to MR examination such as claustrophobia. In addition, for every AMD subject, the age when they first used MA, the total time of MA use, the frequency of MA use in the last month before abstinence, the abstinence duration, and the craving score were recorded.

The research protocol was approved by the Ethics Committee of the Second Xiangya Hospital, Central South University. All subjects volunteered to participate in this study and signed the informed consent form. Confidentiality of personal information and freedom to withdraw from the study were guaranteed.

### MR Imaging Acquisition

All MRI data were acquired on a 3T Siemens Skyra MRI scanner (Magnetom Skyra, Siemens, Germany) equipped with a 32-channel head coil. The MRI scanning included T1-weight imaging (T1WI), T2-weight imaging (T2WI), and fluid attenuated inversion recovery (FLAIR) sequences. Each scan included a high-resolution T1-weighted anatomical magnetically prepared rapid acquisition gradient echo (3D MPRAGE) sequence with the following parameters: TR = 2,000 ms, TE = 2.6 ms, TI = 900 ms, flip angle = 8°, 176 slices, slice thickness = 1 mm, slice spacing = 1 mm, FOV = 256 × 256 mm^2^, acquisition matrix = 256 × 256, voxel size = 1 × 1 × 1 mm^3^. Subjects were placed in a supine position with foam padding between their head and the head coil to minimize head motions.

### Voxel-Based Morphometry (VBM)

The quality of the T1-weighted images was visually checked for artifacts, structural abnormalities, and apparent head motion and no subject was excluded. Images were processed with Voxel-based Morphometry 8 (VBM8) toolbox (http://dbm.neuro.uni-jena.de/vbm/). Brain images were bias corrected, segmented and spatially normalized to the standard Montreal Neurological Institute (MNI) space. To preserve the actual gray matter values locally, segmented gray matter images were then modulated by a procedure in which the intensity value of each voxel was multiplied by the local value of the Jacobian determinants. Finally, the modulated gray matter volume were smoothed with an 8 mm full-width at half maximum (FWHM) Gaussian kernel.

### Statistical Analysis

Demographics were compared between the groups with SPSS 21.0. Patients with AMD and healthy control groups were compared on age and the years of education using two-sample *t*-test; smoking and drinking using chi-square test, and the significance level was set to *p* < 0.05.

Voxel-wise GMV differences between two groups were computed using two-sample *t*-test with individual's total GMV, age, gender, and education as covariates. The group GMV difference was corrected for multiple comparisons to a significant level of *p* < 0.05 by combining the individual voxel *p* < 0.01 and cluster size >603 voxels. This correction was confined within a whole brain mask and was determined by Monte Carlo simulations using the DPABI AlphaSim program. On regions showed significantly different GMV between AMD group and healthy group, partial correlation coefficients adjusted for age, years of education, smoking, and drinking were calculated in AMD group to assess association between the mean GMV difference and their age when they first used MA, the total time of MA use, the frequency of MA use in the last month before abstinence, the abstinence duration and the craving score. Significance level was set to *p* < 0.05.

## Results

### Demographics and Clinical Characteristics of the Participants

Forty-four AMD subjects and forty healthy subjects are included in this study. There is no significant difference between AMD group and HC group in age (mean ± SD) (33.1 ± 6.8 for AMD group; 34.3 ± 7.5 for HC group; *t* = 0.760, *p* = 0.450), the years of education (8.7 ± 2.2 for AMD group; 9.5 ± 2.3 for HC group; *t* = 1.656, *p* = 0.102), smoking (43 of 44 AMD subjects smoke; 38 of 40 HC subjects smoke; χ^2^ = 0.007, *p* = 0.933) and drinking (16 of 44 AMD subjects drink; 8 of 40 HC subjects drink; χ^2^ = 2.006, *p* = 0.157) as showed in Table 1.

**Table 1:** Demographic information and characterization.

**Group**	***N***	**Age/year**	**Education/year**	**Smoke**		**Drink**	
				**Yes**	**No**	**Yes**	**No**
AMD	44	33.1 ± 6.8	8.7 ± 2.2	43	1	6	28
HC	40	34.3 ± 7.5	9.5 ± 2.3	38	2	8	32
		*t* = 0.760	*t* = 1.656	χ^2^ = 0.007		χ^2^ = 2.006	
*p*		0.450^a^	0.102^a^	0.933^b^		0.157^b^	

a *Two-sample t-test*.

b *Chi-square test*.

*Significant level was set at p < 0.05. There are no statistically significant differences between AMD and HC group based on demographic information and characterization. N, number of participants; AMD, abstinent methamphetamine-dependent; HC, healthy control*.

### VBM Results

**Figure 1:**
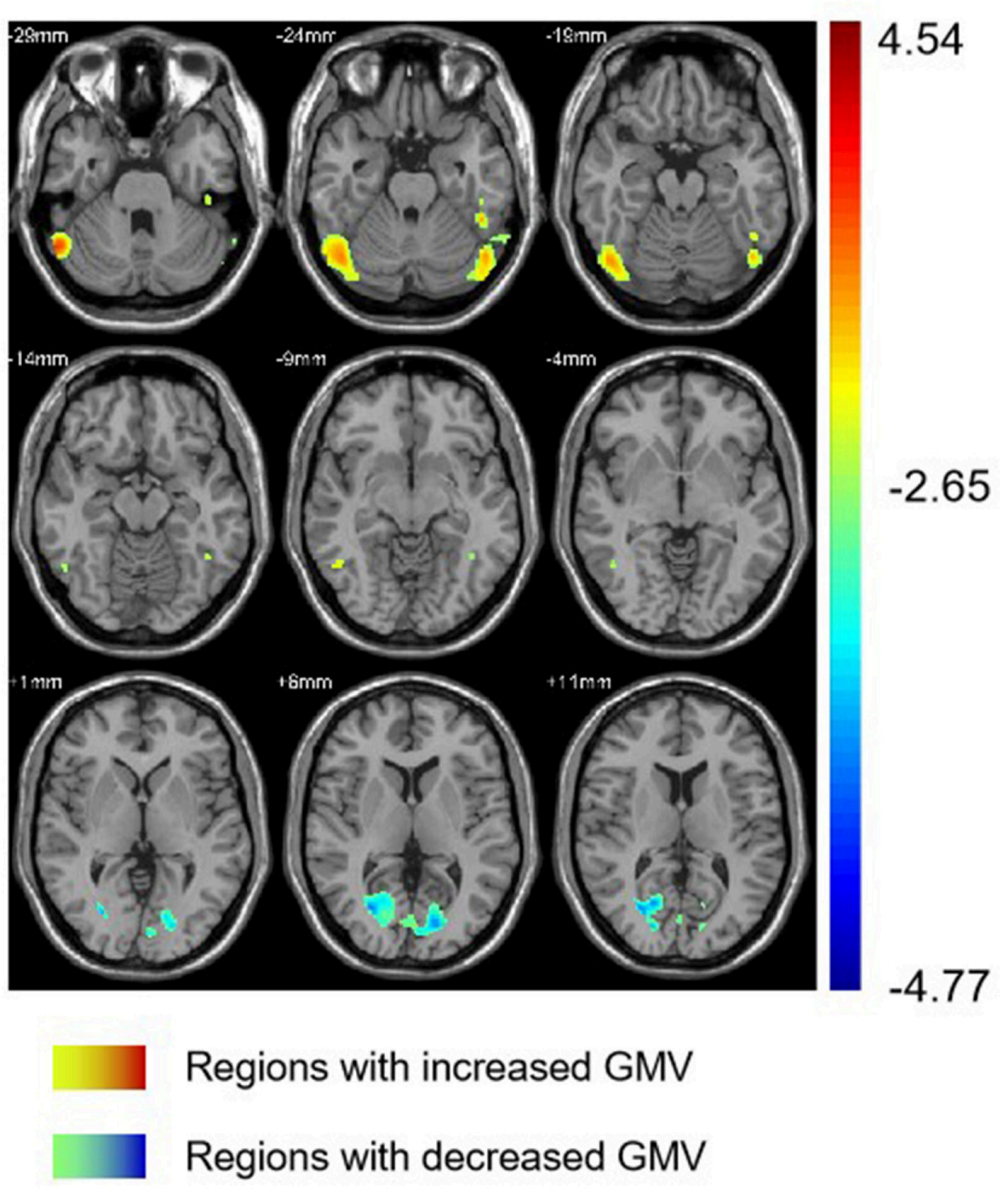
Regions with different GMV in AMD group compared with HC group. GMV, gray matter volume; AMD, abstinent methamphetamine-dependent; HC, healthy control.

Group differences are shown in Tables 2, 3 and Figure 1. In comparison with HC group, the significant GMV reductions in AMD group were around right calcarine and right cuneus. In contrast, the significant GMV increases in AMD group are around the left cerebellum and right cerebellum.

**Table 2:** Regions with reduced GMV in AMD group compared with HC group.

**Brain region (AAL)**	**Peak *t*-value**	**Cluster size (voxels)**	**Peak MNI coordinates**		
			**X**	**Y**	**Z**
Calcarine_R	−3.7848	846	27	−70.5	9
Cuneus_R	−3.7277	1,240	7.5	−75	22.5

*Statistical threshold was p < 0.05 corrected for multiple comparisons by AlphaSim. A combination threshold of voxels' p < 0.01 and cluster size >603 voxels was considered significant; Coordinates are located in Montreal Neurological Institute (MNI) space; L, left hemisphere, R, right hemisphere; AAL, Anatomic-Automatic-Labeling template*.

**Table 3:** Regions with increased GMV in AMD group compared with HC group.

**Brain region (AAL)**	**Peak *t*-value**	**Cluster size (voxels)**	**Peak MNI coordinates**		
			**X**	**Y**	**Z**
Cerebelum_Crus1_L	3.6753	858	−48	−67.5	−22.5
Cerebelum_Crus1_R	3.9336	1,455	58.5	−63	−31.5

*Statistical threshold was p < 0.05 corrected for multiple comparisons by AlphaSim. A combination threshold of voxels' p < 0.01 and cluster size >603 voxels was considered significant; Coordinates are located in Montreal Neurological Institute (MNI) space; L, left hemisphere, R, right hemisphere; AAL, Anatomic-Automatic-Labeling template*.

### Correlation Analyses

**Figure 2:**
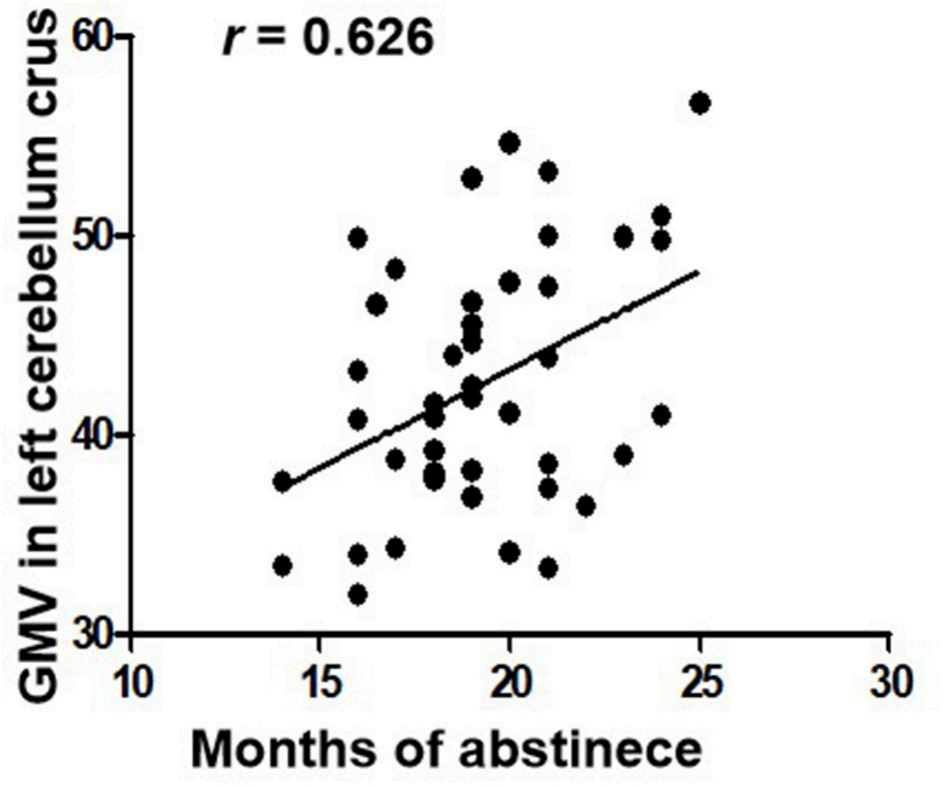
Correlations between duration of abstinence and GMV in the left cerebellum crus in AMD subjects. GMV, gray matter volume; AMD, abstinent methamphetamine-dependent.

The length of abstinent duration of AMD subjects is positively correlated with GMV in left cerebellum crus as showed in Figure 2 (*p* = 0.040, *r* = 0.626). However, no other significant correlation was found in the AMD group.

## Discussion

In our study, we compared GMV between abstinent methamphetamine-dependent group and healthy control group using VBM. We found that AMD group showed significant increased GMV in the bilateral cerebellum crus, and decreased GMV in the right calcarine and right cuneus. In addition, in the AMD group, duration of abstinence is positively correlated with GMV in the left cerebellum crus.

Numerous studies have confirmed drug abuse reduces volume of the cerebellum (31–33). Studies showed that the cerebellum is involved in the procedure of addiction, such as memory, predictive power, and executive control ability (34). Researches have shown that cerebellum crus is associated with cognitive and emotional function (35–37). In our study, the volume of bilateral cerebellar crus was increased in AMD group, which is consistent with previous studies. Kühn et al. (38) confirmed that cerebellar gray matter volume is negatively correlated with the degree of nicotine dependence. Schwartz et al. (39) found that patients with heroin dependence had an increased density of gray matter after 2 months of withdrawal, associated with cognitive, memory, and mood improvements, and with decreased levels of craving for drugs. A spectroscopy study on the abstinence of methamphetamine found that abstinence therapy contributes to the normalization of cerebellar neurometabolites (29). Therefore, we speculated that increased GMV of the cerebellum founded in our study may indicate partial recovery of cerebellar function in AMD patients. Importantly, we also found that the duration of abstinence was positively correlated with the left cerebellar volume, which may suggest that the long-term abstinence is beneficial for cerebellum recovery. Moreover, as the left cerebellar volume GMV increases over abstinence time, we inferred that this increase is probably associated with abstinence instead of addiction.

The cortex around the calcarine fissure is the primary visual cortex, which accepts direct projection from the retina to recognize text, identify objects, determine the relationship between objects, distance difference, recent memory, and so on (40, 41). Neuroimaging studies often report that the activity in visual areas is significantly associated with drug cues exposure, treatment effect of drug abuse and prediction of relapse. It has shown that neural circuitry of addiction, consistently discriminates drug cues from neutral cues in substance dependence. A study by Helenna et al. based on VBM, found that visual associated cortices showed decreasing trends of cortical gray matter volumes on methamphetamine abusers, which may contribute to psychiatric symptoms (42). Our result that the volume of the visual cortex of AMD group decreased implies that drug cue-induced craving, which is one of the most robust factors to continued use and relapse across substances (43), is significant among methamphetamine abuser after long-term abstinence.

In our study, the AMD group also showed decreases in right cuneus volumes. The cuneus is involved in visual processing (44) and associated with cessation outcomes (43). A VBM study (45) on smokers showed that compared to relapsers, quitters had significantly smaller GMV in their right cuneus. Therefore, it is possible that the decreased GMV of cuneus has an impact on abstinence and relapse.

## Strength and Limitations

The current study investigated AMD subjects' brain alterations after long-term abstinence. To the best of our knowledge, this is the first study to reveal brain gray matter alterations after an extended abstinence duration. Changes on visual and cognitive function regions suggest that these areas play important roles in the progress of MA addiction and abstinence.

There are several limitations in this study. (1) This is a cross-sectional study, the image data of AMD subjects before abstinence was not collected. As a result, the causal link of abnormal gray matter volume and the abstinence status was not determined. (2) Most of the subjects and controls have a history of smoking. The effect of smoking on the gray matter volume of the brain has been confirmed in the literature (46). Although smoking status is a covariate in this study, the influence of smoking on brain structure cannot be completely excluded. Further study with non-smoking subgroups would help to address this issue. (3) There are no female subjects in this study. The sex differences in the brain can influence the responses to drugs of abuse, progressive changes in the brain after exposure to drugs of abuse and whether addiction results from drug-taking experiences (47). For example, women exhibit more rapid escalation from casual drug taking to addiction, exhibit a greater withdrawal response with abstinence, and tend to exhibit greater vulnerability than men in terms of treatment outcome (48). Therefore, our results are only applicable in male subjects.

In summary, based on the VBM analysis, this study found gray matter changes in the bilateral cerebellar crus, the right calcarine and right cuneus in methamphetamine-dependent subjects with long-term abstinence. In addition, the volume of left cerebellum was positively correlated with abstinent duration, suggesting that prolonged abstinence may be beneficial to cognitive function recovery. This study provides an imaging basis for revealing the neural mechanism of long-term abstinence of methamphetamine.

## Author Contributions

JL and JZ conceptualized and designed the research. PL, SH, LF, and YL performed the experiments. RY undertook the statistical analysis. ZZ, LH, and YJ wrote the first draft of the manuscript. ZZ and RY contributed to the final manuscript. All authors critically reviewed content and approved final version for publication.

### Conflict of Interest Statement

The authors declare that the research was conducted in the absence of any commercial or financial relationships that could be construed as a potential conflict of interest.
